# Visuomotor and motorvisual priming with different types of set-level congruency: evidence in support of ideomotor theory, and the planning and control model (PCM)

**DOI:** 10.1007/s00426-017-0885-3

**Published:** 2017-07-29

**Authors:** Roland Thomaschke, R. Christopher Miall, Miriam Rueß, Puja R. Mehta, Brian Hopkins

**Affiliations:** 1 Lancaster University, Lancaster, UK; 2 Institut für Psychologie, Albert-Ludwigs-Universität Freiburg, Engelbergerstraße 41c, 79085 Freiburg, Germany; 3 Birmingham University, Birmingham, UK; 4 King’s College London, London, UK

## Abstract

Perception can prime action (visuomotor priming), and action can prime perception (motorvisual priming). According to ideomotor theory both effects rely on the overlap of mental representations between perception and action. This implies that both effects get more pronounced the more features they share. We tested this hypothesis by employing in a motorvisual (Exp. 1) and in a visuomotor (Exp. 2) setting, three different pairs of left/right target stimuli (hand pictures, arrows, and words) varying in how strongly they overlap with the pair of left/right responses. For two stimulus pairs (hands and words) the hypothesis was confirmed: hand pictures share more features with the responses than words, consequently hand pictures produced a stronger visuomotor and a stronger motorvisual priming effect than words. However, arrow stimuli showed a different pattern: the temporal dynamics of both priming effects, as well as the direction of the effect seen in motorvisual priming, were significant but opposite to that of the hand and word stimuli. This suggests that the arrows’ representations were not involved in ideomotor processes, and we propose instead that they were represented in a spatial or scalar fashion, outside the representations assumed in ideomotor theory. The results are discussed in the context of ideomotor theory, and the planning and control model of motorvisual priming.

## Introduction

Action and perception are strongly coupled. Their close entanglement in the human cognitive system results in strong bidirectional influences between perceptual and motor processes. On the one hand, perception has direct automatic effects on action. Stimulus features automatically prime congruent action features, even when these stimulus features are task-irrelevant (Simon & Rudell, 1967). For instance, when discriminating the color of a direction word (i.e., ‘left’ or ‘right’) by left and right button presses, the task-irrelevant word meaning automatically primes the congruent response (i.e., left responses are faster to the word ‘left’, and right responses are faster to the word ‘right’, see, e.g., Pellicano, Lugli, Baroni, & Nicoletti, 2009). Priming effects from perceptual processes on congruent actions are commonly referred to as visuomotor priming (Craighero, Fadiga, Rizzolatti, & Umiltà, 1998). However, priming also works in the reverse direction: actions can affect the perception of congruent stimuli (e.g., Müsseler & Hommel, 1997a; for a review, see Thomaschke, 2012). In a dual task, for example, speaking the words ‘left’ or ‘right’ impair the perception of a congruent word in the other task (i.e., speaking ‘left’ impairs the perception of the word ‘left”, and speaking ‘right” impairs the perception the word ‘right’, see, e.g., Hommel & Müsseler, 2006). Such priming effects (in this case negative priming) from actions on the perception of action-congruent stimuli are commonly referred to as motorvisual priming (Thomaschke, 2012).

Visuomotor and motorvisual priming effects can both be explained by a common conceptual framework: the ideomotor theory. The ideomotor theory claims that actions are represented by their perceivable consequences. Put in another way, action selection processes and perceptual processes operate on a shared pool of representations (Amer, Gozli, & Pratt, 2017; Hommel, Müsseler, Aschersleben, & Prinz, 2001; Prinz, 1997; Shin, Proctor, & Capaldi, 2010). Consequently, the selection of an action and the perception of an action-congruent stimulus mutually influence each other.

However, ideomotor theory predicts positive priming effects for visuomotor priming and negative priming effects for motorvisual priming. In visuomotor priming, the perception of a stimulus automatically activates representations (e.g., for the feature “left”) that are also involved in selecting congruent (i.e., including the feature “left”) actions. Thus, actions to congruent stimuli are selected based on pre-activated representations, and are, consequently, faster and less error-prone than actions to incongruent stimuli (Proctor & Vu, 2006).

Motorvisual priming, on the contrary, is negative. It usually occurs when, in a dual-task situation, actions in one task share a feature (e.g., “left”) with stimuli in the other task. The priming is negative, because already selected action features are bound together with other features of the selected action into a common event file (Hommel, 2004). Features in an event file are thought to be shielded against all other concurrent cognitive processes, including perceptual processes. Thus, when selecting an action with a certain feature (e.g., “left”) in one task, perceptions of stimuli including congruent features (e.g., “left”) in another task are impaired (e.g., Eder & Klauer, 2007, 2009; Gozli & Pratt, 2011; James & Gauthier, 2009; Kunde & Wühr, 2004; Müsseler & Hommel, 1997b).

When, however, visuomotor and motorvisual priming are really both based on shared representations between perception and action, both phenomena should get more pronounced, the more features are shared between stimuli and responses. For visuomotor priming, the more action features are pre-activated by the stimulus perception, the more action selection should be facilitated. For motorvisual priming, the more stimulus features are occupied and shielded by action selection, the more stimulus perception should be impaired. This prediction can more precisely be formulated in the terminology of element-level and set-level congruence.

### Element- and set-level congruency

Visuomotor and motorvisual priming experiments typically involve a set of stimulus elements (e.g., a left or right pointing arrowhead), as well as a set of response elements (e.g., a left or a right button press). Element-level congruence refers to the match or mismatch between the particular stimulus element and the particular response element on a given trial. For example, a left pointing arrowhead is element-level congruent to a left button press. The visuomotor and motorvisual priming effects described above are congruency effects on the element level: positive priming means element-congruent trials yield better performance than element-incongruent ones (as is typically found with visuomotor priming, see above). Negative priming, on the other hand, means incongruent trials yield better performance than congruent ones (as is typically found with motorvisual priming). The magnitude of an element-level congruency effect is defined as the performance difference between element-congruent and element-incongruent trials (Thomaschke, Hopkins, & Miall, 2012b).

Set-level congruence, on the contrary, refers to the degree of shared feature dimensions between the stimulus set and the response set. Consider, for example, a stimulus set comprising photographs showing a left or a right button press, and a response set comprising a left or a right button press (e.g., Brass, Bekkering, Wohlschläger, & Prinz, 2000). These sets have relatively high set-level congruence, because they overlap not only on an abstract symbolic “left/right” dimension, but also on a variety of low-level physical anatomical dimensions. Put simply, button presses and pictures of button presses are highly similar to each other (see Miles & Proctor, 2012; Proctor & Vu, 2006, for a detailed review and discussion of overlap dimensions).

Other sets have relatively low set-level congruence. Consider the stimulus set comprising the written words LEFT and RIGHT, and the response set comprising a left or right button press. These sets overlap on only one dimension: the abstract semantic category ‘left’/‘right’. Thus, the set-level congruence of this pair of sets is lower than the set-level congruence of the former pair of sets. Put simply, direction words and button presses are only moderately similar to each other.

### Ideomotor theory and set-level congruency

In terms of set- and element-level congruency, ideomotor theory’s prediction concerning visuomotor and motorvisual priming can be formulated as follows: for both—visuomotor and motorvisual priming—element-level congruency should increase with set-level congruency. This means, for visuomotor as well as for motorvisual priming, the priming effects should get stronger the more feature dimensions are shared between the stimulus set and the response set.

Consider visuomotor priming first. When more feature dimensions overlap between stimulus and response, more features of an action are pre-activated in congruent trials. This leads to a bigger advantage over incongruent trials (i.e., to a stronger element-level congruency effect). Thus, a pair of sets with high set-level congruency (e.g., button presses and pictures of button presses) should produce a stronger element-level congruency effect, than a pair of sets with low set-level congruency (e.g., button presses and written direction words).

The same applies to motorvisual priming. The more features are shared between an action and a concurrently displayed stimulus the more perceptually relevant features are bound into the action’s event file. Consequently, the perceptual processing of more features is impaired in congruent trials. Thus, with higher feature overlap between action and perception, the perceptual impairment in congruent relative to incongruent trials should be stronger. Higher set-level congruency should lead to more pronounced negative priming effects on the element level.

The influence of set-level congruency on the magnitude of element-level congruency effects has been frequently studied for visuomotor priming (e.g., Miles & Proctor, 2012, for an overview). For motorvisual priming, on the contrary, there is only sparse knowledge about the relation between set- and element-level congruency, and we know of only one published study looking at this (Hommel & Müsseler, 2006).

In the present study, we have two aims: first, confirming earlier findings that set-level congruency increases the element-level congruency effect for visuomotor priming, as well as for motorvisual priming. Second, we go beyond previous studies by doing this with exactly the same stimulus material for both visuomotor and motorvisual effects. This enables us to test whether the same set-level and element-level relations are obtained for both effects. We hypothesize that when one pair of sets A yields a stronger visuomotor priming effect than another pair of sets B, then A should also yield a stronger motorvisual priming effect than B, and vice versa. It was not possible to infer this relation directly from previous studies, because no previous study has investigated motorvisual and visuomotor effects in the same set of experiments. The stimulus and response sets in published studies on the effects are either constructed in different ways, or the reports lack of sufficient detail to precisely estimate their similarity between the employed stimulus sets across studies. Thus, it is not possible to estimate whether a stimulus set, for instance, of left and right pointing arrowheads, has a comparable set-level congruence with, for instance, button press responses in different studies. Before introducing the design of our study in more detail, we briefly review previous research on set-level effects on visuomotor and motorvisual priming.

### Set-level congruency in visuomotor priming

Priming effects from irrelevant stimulus features on responses—typically referred to as Simon effects—have been investigated by a substantial number of studies (reviewed in Hommel, 2011; Proctor, 2011). These studies have employed various different stimulus and response sets. In the following we mainly focus on the stimulus and response sets similar to the ones employed in the present study, that is arrows, direction words, and hand pictures, paired with horizontally aligned button presses.

The majority of studies involving more than one of these stimulus sets employed arrows (i.e., pointing left/right) and words (i.e., ‘left’/‘right’) as stimulus sets, showing, however, mixed results. Testing the Simon effect for arrows and for words in different experiments, Pellicano et al. (2009) observed the same effect magnitude (22 ms) for both stimulus pairs. These results are in line with a study by Miles and Proctor (2012). They compared arrows and words in different blocks within subjects (Exp. 1). Although the Simon effect was numerically larger for arrows (32 ms) than for words (27 ms), no significant interaction between congruency and stimulus set was observed. However, in a second experiment, Miles and Proctor mixed stimulus sets randomly within blocks of trials. Under this condition, arrows showed a significantly smaller Simon effect (21 ms) than words (44 ms). Yet, a significant difference in the opposite direction was observed in a study by Proctor, Yamaguchi, Zhang, and Vu (2009). In between-subjects comparisons, arrows produced stronger Simon effects (44 ms, Exp. 1; 47 ms, Exp. 3) than words (28 ms, Exp. 1; 20 ms, Exp. 2). However, it is not clear whether this difference is reliable, because the data were collapsed across conditions, where the Simon experiment was preceded by different learning procedures with location classification tasks.

The only relevant study also employing hand pictures is due to Kornblum and Lee (1995). It involved one condition where participants responded by finger presses to letters. The letters were displayed on either response-congruent or response-incongruent fingers of a drawing of a pair of hands. Another condition required vocal letter responses to fingers marked by letters on the same drawn hands. The irrelevant identity of the marking letter was either congruent or incongruent to the vocal response. Irrelevant letters produced a slightly smaller Simon effect (47 ms, Exp. 2; 53 ms, Exp. 3) than did irrelevant finger identity (52 ms, Exp. 2; 55 ms, Exp. 3), but the effects have not been statistically compared with each other.

In summary, the results concerning the influence of set-level congruency on the Simon effect are varied, even pointing in different directions. On the one hand, these inconsistencies might be due to complex modulations of the Simon effect by experimental contexts. On the other hand, they might be merely due to idiosyncratic differences in the stimuli used in the different studies. For example, Pellicano et al. (2009, Exp. 1) found that the shape of the arrow stimuli (greater than/less than symbols vs. outline drawings of proper arrows) tended to affect Simon effect magnitude. Likewise, the discriminability of written stimulus words affects the Simon effect (Miles & Proctor, 2009). This means that comparisons between previous Simon-effect studies are problematic, as they are very sensitive to the shape, size, and salience of the employed stimulus sets.

### Set-level congruency in motorvisual priming

Previous motorvisual priming studies have applied many different stimulus sets and response sets, overlapping on various dimensions, ranging from gesture identity (Miall et al., 2006; Stanley & Miall, 2009, Yon & Press, 2017), over movement type (Jacobs & Shiffrar, 2005), movement direction (Zwickel, Grosjean, & Prinz, 2007, 2010; Zwickel & Prinz, 2012), left/right categorization (Müsseler & Hommel, 1997a, b), number (Kunde & Kiesel, 2006), object size (Symes, Tucker, Ellis, Vainio, & Ottoboni, 2008), object weight (Hamilton, Wolpert, & Frith, 2004) orientation (Lindemann & Bekkering, 2009; Pfister, Heinemann, Kiesel, Thomaschke, & Janczyk, 2012), letter form (James & Gauthier, 2009), and color (Kunde & Wühr, 2004), to affect (Eder & Klauer, 2007, 2009).

Despite the large number of different stimulus and response sets employed in motorvisual priming, there is virtually no previous study comparing different pairs of sets in one study. Thus, knowledge about set-level congruency effects in motor visual priming is extremely scarce (Hommel & Müsseler, 2006). Response sets in that study were key presses (left and right) and spoken words (left and right). Stimulus sets were arrows (pointing left and right) and printed words (“left” and “right”). Motorvisual priming effects were observed only when set-level congruency was high (i.e., printed words with spoken words, and arrows with key presses), but not when it was low (i.e., printed words with key presses, and arrows with spoken words). This pattern of results suggests that, as for visuomotor priming, motorvisual effects of element-level congruency increase with set-level congruency.

### Aim of the present study

We analyze the effects of set-level congruency on visuomotor priming and on motorvisual priming, for the first time in one study employing identical stimulus sets to access both priming directions. To this end, we employ three different stimulus sets (finger pictures, arrows, and words) differing in their set-level congruency to the response set (left/right button presses).

We hypothesize that effect magnitude will increase with higher set-level congruency for visuomotor priming as well as for motorvisual priming. That is, priming effects will be stronger for finger pictures than for arrows than for words, and this will be the case for positive priming in a visuomotor experiment as well as for negative priming in a motorvisual experiment.

## General method

### Overview

We conducted one motorvisual priming experiment (Exp. 1) and one visuomotor priming experiment (Exp. 2). The motorvisual experiment was realized as a dual task, the most common motorvisual priming procedure (Thomaschke, 2012). In this procedure, participants had to do two independent tasks in each trial—a motor task and visual discrimination task. In the motor task, they had to respond with a left or right key press to a symbolic cue. In the visual discrimination task they had to identify a masked target stimulus, and report it later by another key press. Importantly, the visual task’s target stimulus was displayed during the motor task’s response, so that motor processing in the motor task could impair visual processing in the visual discrimination task. In order to control the strength of this impairment, we manipulated the congruency between the motor task’s response and the visual task’s target stimulus. In congruence trials, for example a left target had to be discriminated during a left key press, whereas in incongruent trials, for example, a right target had to be discriminated during a left key press. A motorvisual priming effect would be realized here as worse discrimination in congruent relative to incongruent trials.

The visuomotor experiment was realized as a classic Simon paradigm with stimulus–response overlap on different dimensions. Participants had to respond by left and right key presses to the color of centrally presented stimuli, while the stimuli also conveyed task-irrelevant left/right information.

In both tasks, we compared priming effects with three different stimulus pairs. In order to allow comparisons between set-level congruency effects in visuomotor and motor visual priming, we employed the same stimulus sets as visual discrimination targets in Experiment 1 and as imperative stimuli in Experiment 2.

### Apparatus

**Fig. 1:**
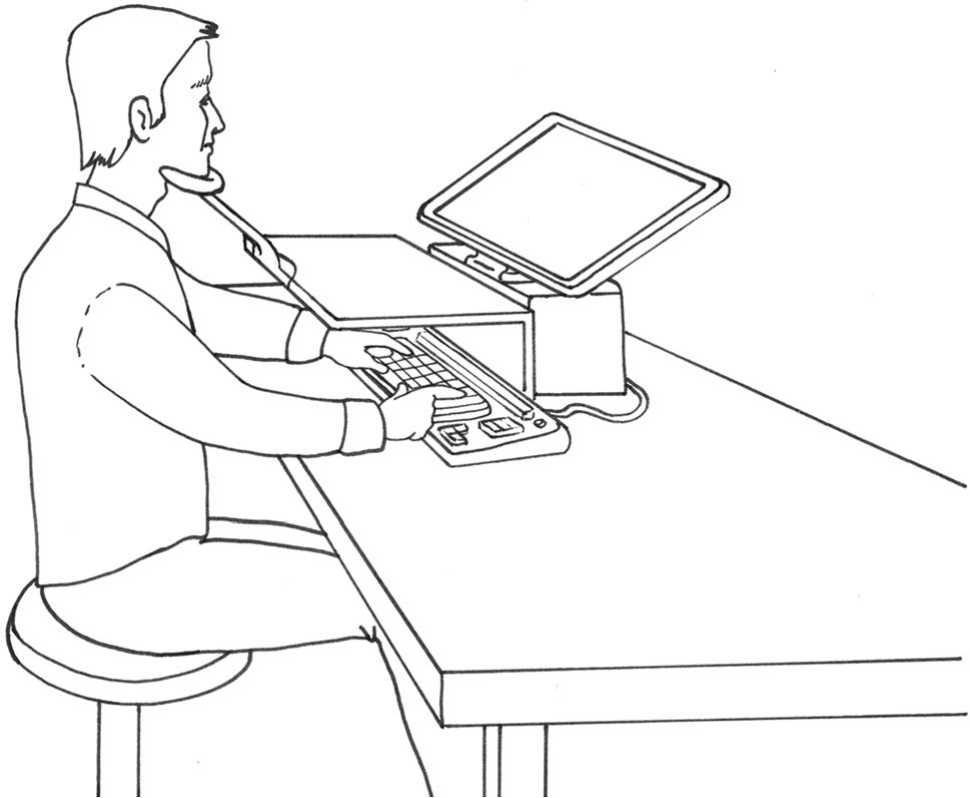
Apparatus. Participants’ head position was adjusted by a chin rest, with the hands occluded form view

Both experiments were conducted in a dimly lit room. Participants sat at a desk in front of a computer screen and a keyboard. The viewing distance and viewing angle were adjusted by a chin rest such that the screen surface was perpendicular to their viewing direction at a distance of about 50 cm from their eyes. The index fingers of both hands rested on the adjacent keys ‘b’ (left index finger) and ‘n’ (right index finger) of a USB connected Mac OS key board (British Standard layout; BS 4822). The keyboard was occluded from the participants’ view by a horizontal plane below the screen (see Fig. 1).

The experiments were run on an Apple G4 computer and monitor. The monitor had a screen refresh rate of 60 Hz. Consequently, the term ‘screen cycle’ refers, in the remainder of this paper, to a time interval of about 16.7 ms. Stimuli were displayed with a resolution of ca. 38 pixel per cm. Stimulus display, response measurement, and data collection were controlled via the Psychophysics Toolbox extension (Version 3) of the Matlab software (Brainard, 1997; Pelli, 1997).

### Stimuli

**Fig. 2:**
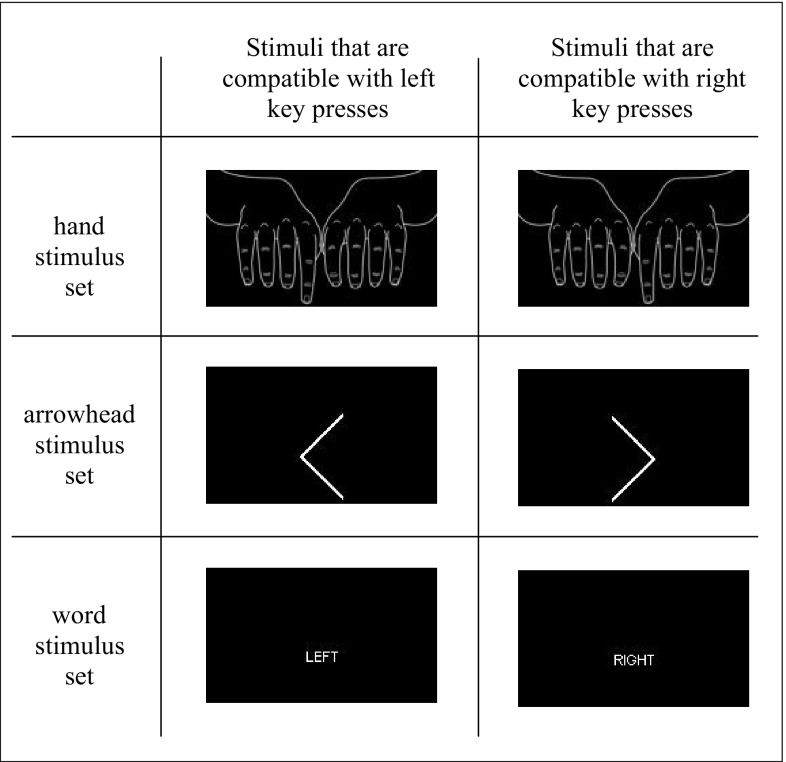
Stimulus sets. The three stimulus sets are displayed in different contrasts. The hand stimulus set is shown with a contrast value of 0.6, the arrowhead stimulus set has a contrast of 0.9, and the word stimuli are rendered with a contrast value of 0.8

For the sake of comparability between this and other studies and between different stimuli sets within this study, we constructed the stimuli in a way that the choice-relevant parts of the figures occupied about the same area, and involved an approximately equal number of pixels. The occupied area was comparable to previous priming studies with those stimuli. Despite these similarities, the figure did—necessarily—still differ in many structural aspects (see Fig. 2). All stimuli were displayed in the screen center on a constant black background.

#### Hands

The hand stimuli were line drawings, depicting the end positions of a left and a right key press movement (see Fig. 2). The left and right stimulus both consisted of a drawing of a pair of hands in a position as if they were resting next to each other on a keyboard. The pictures did not include any objects (e.g., keyboard, or any surface) besides the hands. The left stimulus picture was precisely mirror symmetric to the right one with regard to the vertical axis. The only difference between the stimuli was that in the left stimulus the index finger of the left hand (left, from the participant’s perspective) was extended as if pressing a key on a keyboard, and in the right stimulus the index finger of the right hand was extended. End positions of movements (key presses) were chosen in order to maximize priming effects. Stürmer, Aschersleben, and Prinz (2000) found that still pictures of movement-end positions show stronger compatibility effects than still pictures of intermediate positions, or movie displays of full movements. White on black line drawings were chosen, instead of photographs, because the color homogeneity of line drawings allowed one to manipulate the contrast in a way that was comparable with the other two stimulus sets (see “Procedure” of Experiment 1).

One might speculate that the participants would actually process the hand displayed on the left (from their perspective) as congruent to their own right hand, because the hand displayed on the left would be the right hand of an imagined individual (considered from the individual’s perspective) sitting opposite to the participant. However, a substantial number of studies in the area of imitation priming has investigated congruence between own and displayed hands in different perspectives (e.g., Brass, Bekkering, Wohlschläger, & Prinz, 2000; Press, Bird, Walsh, & Heyes, 2008; Vogt, Taylor, & Hopkins, 2003). These studies unanimously show that ipsilateral side is more important than anatomical identity for congruence between own and displayed hands (e.g., Bertenthal, Longo, & Kosobud, 2006; Sutter & Müsseler, 2010).

In order to achieve comparability between the stimulus sets with regard to pixel number and occupied size, only the distinction-relevant area of the hand stimuli was considered for comparison. This is the area occupied by both index fingers from root knuckle to tip (when extended). The rest of the picture is identical between both stimuli, but cannot be omitted, because for a maximal set-level congruency it is important that participants automatically recognize the crucial index finger areas in their anatomical context (i.e., as two index fingers of adjacent hands).

The distinction-relevant area included 2048 pixels (width = 32 pixel, height = 64 pixel). The white parts of the figure in this area consisted of 203 pixels for each of the stimuli. The overall size of the relevant area (0.97° × 1.94° of visual angle) is comparable to the majority of previous visuomotor and motorvisual priming studies.

#### Arrows

The arrows were similar to the “smaller/greater than” symbols employed in the majority of all arrow priming studies with an angle of 45° between both lines (see Fig. 2). The bounding rectangular area was identical with the distinction-relevant area for the hand stimuli (width = 32 pixel, height = 64 pixel). In order to make the total amount of pixels comparable with the hand stimuli, the linewidth was increased along the inner border of the figure. This resulted in an arrowhead figure with a total of 186 white pixels.

#### Words

The word stimuli consisted of the words ‘left’ and ‘right’ written in capital letters using the font ‘Arial’ in a standard height/width proportion and spacing. The word ‘left’ spanned an area of 32 × 58 pixels, ‘right’, spanned 32 × 62 pixels. We chose not to stretch the shorter words to the 32 × 64 pixel area used for the other stimuli, but instead retained their standard height/width proportion in the middle of the area. The linewidth of each letter was homogenously thickened until the total number of used pixels was comparable with the other stimulus sets (see Fig. 2). The resulting words appear slightly bolder than normal. The word ‘right’ had 197 pixels, while the word ‘left’ had 178 pixels.

Specific details of the methods for Experiment 1 and 2 are given below.

## Experiment 1

We compared the classical motorvisual dual-task priming paradigm with three different stimulus sets as target stimuli in the visual discrimination task: hand pictures, arrows and words. According to previous findings with motorvisual priming, we expected negative priming for each stimulus set: that is, we expect better performance when response in the motor task and stimulus in the visual discrimination task are incongruent on the element-level than when they are congruent. The left/right representation should be occupied by motor response processing, and should, consequently, be difficult to access for perceptual processing of a congruent visual discrimination target.

Importantly, we hypothesized that the negative priming effect should be stronger for hand pictures than for arrows, than for words. Words overlap with button presses only on the verbal semantic ‘left’/‘right’ dimension. Arrows overlap with button presses additionally on the non-verbal symbolic level. Finally, pictures of hand movements overlap with hand movements above the verbal and semantic dimensions also on a variety of low-level physical anatomical dimensions.

### Methods

#### Participants

For Experiment 1, half of the participants were students of Lancaster University, the other half were students of Birmingham University. They received £24 or course credit. All participants reported having normal, or corrected-to-normal vision. Eighteen of the 22 participants were female, 16 were right handed. Their mean age was 19.08 (SD 1.70; range 18–26). The sample size of 22 was chosen, because comparable sample sizes did provide robust motorvisual and visuomotor priming effects in previous studies (see, e.g., Thomaschke et al., 2012b).

#### Stimuli

The cues for the motor task’s response were left and right arrowheads. These arrowheads were the same as the ones used as visual discrimination targets in the “arrow” condition (see “General method”). These cues were the same for all experimental conditions and were compatibly mapped to the motor responses (see e.g., Hommel & Müsseler, 2006; Müsseler, 1999).

The motor responses were prompted by color change of a frame. For a certain interval, in each trial, a rectangular frame was displayed. During this interval, the frame changed its color from white to red, and back to white again. The frame circumscribed the stimulus area for the target stimuli in the visual discrimination task (arrow and word stimuli and the distinction-relevant area for hand stimuli, see “General method”). Hence, the frame’s interior measures were 32 pixels (width) and 64 pixels (height). The frame’s border was 3 pixels thick.

The target stimulus sets for the visual discrimination task were the hand picture, arrow, and word sets described in the “General method”.

The visual discrimination targets were followed by a mask. The mask had the same extension as the white/red frame (38 × 70 pixels). Half of the mask’s pixels was black, the other half had the same brightness as the preceding stimulus (see below). Which pixels were grey and which ones were black was determined randomly before each trial.

The report of the visual discrimination was cued by two white question marks measuring together 78 pixels (width) and 133 pixels (height). All error messages were written in black, surrounded by white boxes on the black background.

#### Trial structure

**Fig. 3:**
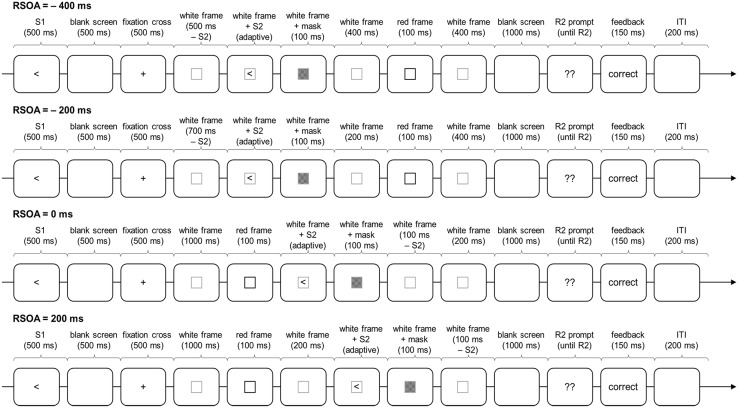
Trial structure for different RSOAs. S1 refers to the response cue, R1 refers to the response. The RSOA refers to the interval between the red frame (go-signal for R1) and display of the target stimulus S2. R2 refers to the report of S2

Each trial began with the display of the cue for the motor response for 500 ms, followed by a black screen for 500 ms, and the fixation cross for another 500 ms (see Fig. 3). During that period participants should have prepared the cued motor response (left or right button press) and keep it on hold. Then, the white frame was displayed and turned red after 1000 ms for only six screen cycles (ca. 100 ms), and white again for further 400 ms. The motor response had to be executed within these six screen cycles where the frame was red.

At some point during the frames, the visual discrimination target was displayed with the frame. Trials differed in response stimulus onset asynchrony (RSOA). This is the time interval between the go-signal for the motor response (frame turning red) and the onset of the discrimination target display. This variable was manipulated in most previous motorvisual priming studies, with contrasting findings concerning the time course of the effect (e.g., Oriet, Stevanovski, Jolicoeur, & Cowan, 2003; Wühr & Müsseler, 2001). The variable has been included in the present experiment in order to test whether potential differences between the three stimulus sets are specific to a certain temporal relation between the motor task’s response and the discrimination task’s target stimulus. Frequently tested RSOAs are −400, −200, 0, and 200 ms (e.g., Hommel & Müsseler, 2006). These RSOAs have also been applied in the present study. Each of the four intervals separated response and stimulus in one quarter of all trials. A negative RSOA means that the target stimulus onset preceded the go-signal for the response (frame turning red, see above). The target stimulus was displayed for a duration that was individually determined before each block (see below) by evaluating visual discrimination performance in the previous blocks. It was immediately followed by the mask for 100 ms.

The frame displays were followed by a black screen for 1000 ms, and then the two question marks. These stayed on the screen until the report for the visual discrimination task was given. The report was given by left or right button presses. These button presses were, in contrast to the earlier motor response, not time-pressured. The report was immediately followed by a written feedback message for 150 ms, saying “correct” or “incorrect”. Trials were separated by a 200-ms interval between the offset of the feedback message in one trial and the onset of the cue for the next trial.

Trials differed along three dimensions: motor response (left, or right), discrimination target (left, or right), and RSOAs (−400, −200, 0, or 200 ms). Consequently, there were 16 (2 × 2 × 4) different trial types. Each of the trial types appeared 16 times as experimental trial in each experimental session. The 256 experimental trials in a session were randomized.

#### Procedure

The experiment was conducted in four separate sessions for each participant. The sessions were conducted on different days with no more than 2 days between two consecutive sessions for each individual. The purpose of the first session was to determine the individually appropriate display times for each participant for each stimulus set. This session will be referred to as the adaptation phase. The three remaining sessions were identical, with the exception that each of them applied a different stimulus set for the visual discrimination targets. The order of the three stimulus sets was counterbalanced across participants. The three latter sessions will be referred to as the experimental phase.

##### Experimental phase

Each session of the experimental phase comprised 18 blocks, the first two of which were practice blocks and were not analyzed. The total duration of a session lasted 65 min. Each block consisted of one practice trial and 16 experimental trials and. The practice trials were not analyzed. Participants paused for 35 s between blocks. An additional break of 3 min was scheduled between the 10th and 11th blocks.

Any invalid trials were repeated at the end of the respective block. When there were more than four invalid trials within one block, the block was not analyzed, and was repeated at the end of the session. The following types of mistakes made a trial invalid:

- The motor response was wrong, meaning it differed from the one that had been, by instruction, assigned to the respective cue.
- The motor response was executed too early, meaning before the respective go-signal (see above).
- The motor response was executed too late, meaning after the go-signal had expired (see above).

Invalid trials or blocks were followed immediately by a specific error message and information that the respective trial or block will be repeated later. Error messages were displayed for 5 s.

##### Adaptation algorithm

The display time for the visual discrimination target was individually adapted throughout the experiment. When a participant judged more than 14 of the 16 targets in a block correctly, the display time was reduced by 1 screen cycle from the consecutive block on. When a participant judged, on the contrary, more than six targets incorrect, the display time was prolonged by one screen cycle. For each experimental session, the initial display time was set to the duration that was determined in the adaptation session for each participant and stimulus set. The initial display time in the adaptation session was three screen cycles for each of the stimulus sets (see below).

In order to make the three stimulus sets comparable with each other also with regard to display time, a second adaptation algorithm, regarding target brightness, was implemented. It was applied only after blocks that did not require an adaptation of display time (i.e., where participants had judged more than 10 and less than 15 stimuli correctly). When this was the case, and when also the display time for the current stimulus set was longer than the display times of both other stimulus sets, then the brightness of the current stimulus set was increased. In the long run, this had the effect that the participant made fewer incorrect judgements for the current stimulus, and that, consequently, its display time was changed, by the primary adaptation algorithm, towards the display times for the other stimulus sets. Likewise, when both other stimulus sets had longer display times, and when the display time of the current stimulus set was not changed after the current block, its brightness was reduced. Brightness increased or reduced in steps of 10% of the full range (0–255), simultaneously in all three Red Green Blue (RGB) channels. Initial brightness for the stimulus sets were 50% for hand and word stimuli, and 30% for arrow stimuli, relative to full brightness (RGB = 255, 255, 255). Pilot studies have shown that these brightness proportions lead to relatively homogeneous display times for the three stimulus sets. Both algorithms were effective throughout the full experiment, including the adaptation session and the practice blocks in the experimental session.

The brightness adaptation was included to prevent the display time adaptation from yielding very different display times, thereby avoiding potential display time effects on cognitive processing.

##### Adaptation phase

The adaptation session differed from the experimental sessions in two main ways. First, the motor response task was absent in the adaptation session. Consequently, the cue for the motor response was not displayed in the adaptation session. However, with the exception of this difference, the trial structure exactly resembled the trials in the experimental sessions. Thus, the go-signal for the motor response was displayed, but had no imperative function. Participants had been informed that it will become relevant in the consecutive experimental sessions. Secondly, all three stimulus sets appeared already in the adaptation session in the same order as they later appeared, one per session, in the experimental phase.

The adaptation session was 15 blocks long—5 for each stimulus set. Each block consisted of 16 randomly ordered trials. Participants paused for 30 s between the blocks, and for an additional 3 min between every fifth block. The purpose of the adaptation session was to determine the individual display times in advance of the experimental sessions. The total duration of the adaptation session was approximately 40 min.

### Results

One participant did not complete all blocks of the fourth session, and was hence excluded from all analyses.

#### Display durations and invalid trials

Table 1 shows the average display times for the first block of each experimental session compared with the average display times of all remaining blocks in the respective experimental session. The relatively small differences show that much of the individual display-time-adaptation had been achieved by the adaptation session. Thus, the differences between display times in individual blocks did not add much variance to the motorvisual priming effect.

**Table 1:** Display times in the experimental phases of Experiment 1

	Average display time in the first block of each experimental session	Average display time in blocks 2–16 of each experimental session
Hand		
Stimuli	1.27 sc	1.09 sc
Arrowhead		
Stimuli	2.17 sc	2.30 sc
Word		
Stimuli	1.65 sc	1.83 sc

The average display time in screen cycles (sc) is given for the first and for the 15 remaining blocks of each experimental session

Participants produced on average 8.4 (SD 4.3) invalid trials for arrow stimuli, 7.6 (SD 3.6) invalid trials for hand stimuli, and 6.1 (SD = 3.9) invalid trials for word stimuli. A *χ* ^2^ test of independence between validity and congruency of trials was conducted separately for each stimulus set, but with no significant results.

#### Mean accuracy

Mean accuracy scores were calculated separately for congruent and for incongruent response–stimulus pairings, for each stimulus set, and for each RSOA (see Table 2). We conducted a three-way ANOVA with the factors stimulus set (hands, arrows, words), RSOA (−400, −200, 0, 200), and congruency (congruent, incongruent). We found main effects for stimulus set, *F*(2, 40) = 4.314, *p* = .020, *η*_*p*_^2^ = 0.177, for RSOA, *F*(3, 60) = 4.970, *p* = .004, *η*_*p*_^2^ = 0.199, but not for congruency, *F*(1, 20) = 0.012, *p* = .913, *η*_*p*_^2^ = 0.001. RSOA interacted with congruency, *F*(3, 60) = 4.121, *p* = .010, *η*_*p*_^2^ = 0.171, and, most importantly, stimulus set also interacted with congruency, *F*(2, 40) = 10.093, *p* < .001, *η*_*p*_^2^ = 0.335. Neither the interaction between RSOA and Stimulus set, *F*(6, 120) = 1.645, *p* = .141, *η*_*p*_^2^ = 0.076, nor the three-way interaction attained significance, *F*(6, 120) = 0.809, *p* = .565, *η*_*p*_^2^ = 0.039.

**Table 2:** Mean accuracy rates for each combination of RSOA, congruency, and stimulus set in Experiment 1

	Hands		Arrows		Words	
	Incongruent	Congruent	Incongruent	Congruent	Incongruent	Congruent
RSOA						
−400	77 (15)	72 (15)	63 (16)	76 (9)	70 (10)	73 (11)
−200	79 (11)	72 (17)	66 (12)	75 (11)	72 (9)	73 (13)
0	78 (13)	69 (15)	71 (13)	76 (8)	72 (13)	73 (10)
200	85 (10)	76 (12)	77 (10)	76 (12)	74 (11)	72 (10)

SDs are displayed in parentheses. Values are rounded to the nearest integer

**Fig. 4:**
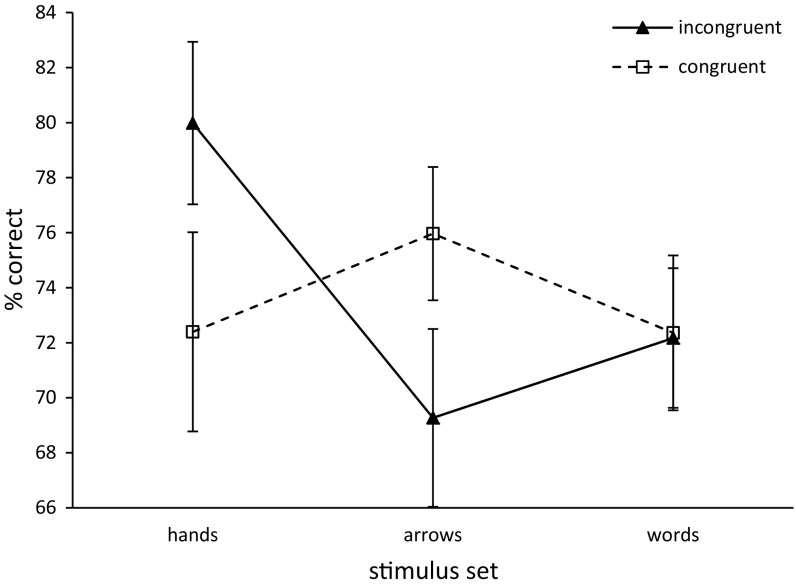
Mean accuracy in Experiment 1. Mean accuracy is displayed in dependence on stimulus set, and congruency. *Error bars* represent inferential confidence intervals, according to Tryon (2001). Non-overlap of a pair of intervals is exactly equivalent to significance at an alpha-level of 0.05 in a within subjects *t* test for congruency

The interaction between stimulus set and congruency was due to motorvisual priming effects in different directions for different stimulus sets: for hand stimuli, performance was significantly better in incongruent trials, *t*(20) = 2.407, *p* = .026, but with arrow stimuli, performance was significantly better in congruent trials, *t*(20) = 2.471, *p* = .023. With word stimuli, the difference between congruent and incongruent trials was not significant, *t*(20) = 0.073, *p* = .943. The priming effect (i.e., performance in congruent trials subtracted from the performance in incongruent trials) differed significantly in pairwise comparisons between all three stimulus sets, *t*(20) = 4.257, *p* < .001, for hands vs. arrows, *t*(20) = 2.426, *p* = .025, for hands vs. words, and *t*(20) = 2.185, *p* = .041 (see Fig. 4).

**Fig. 5:**
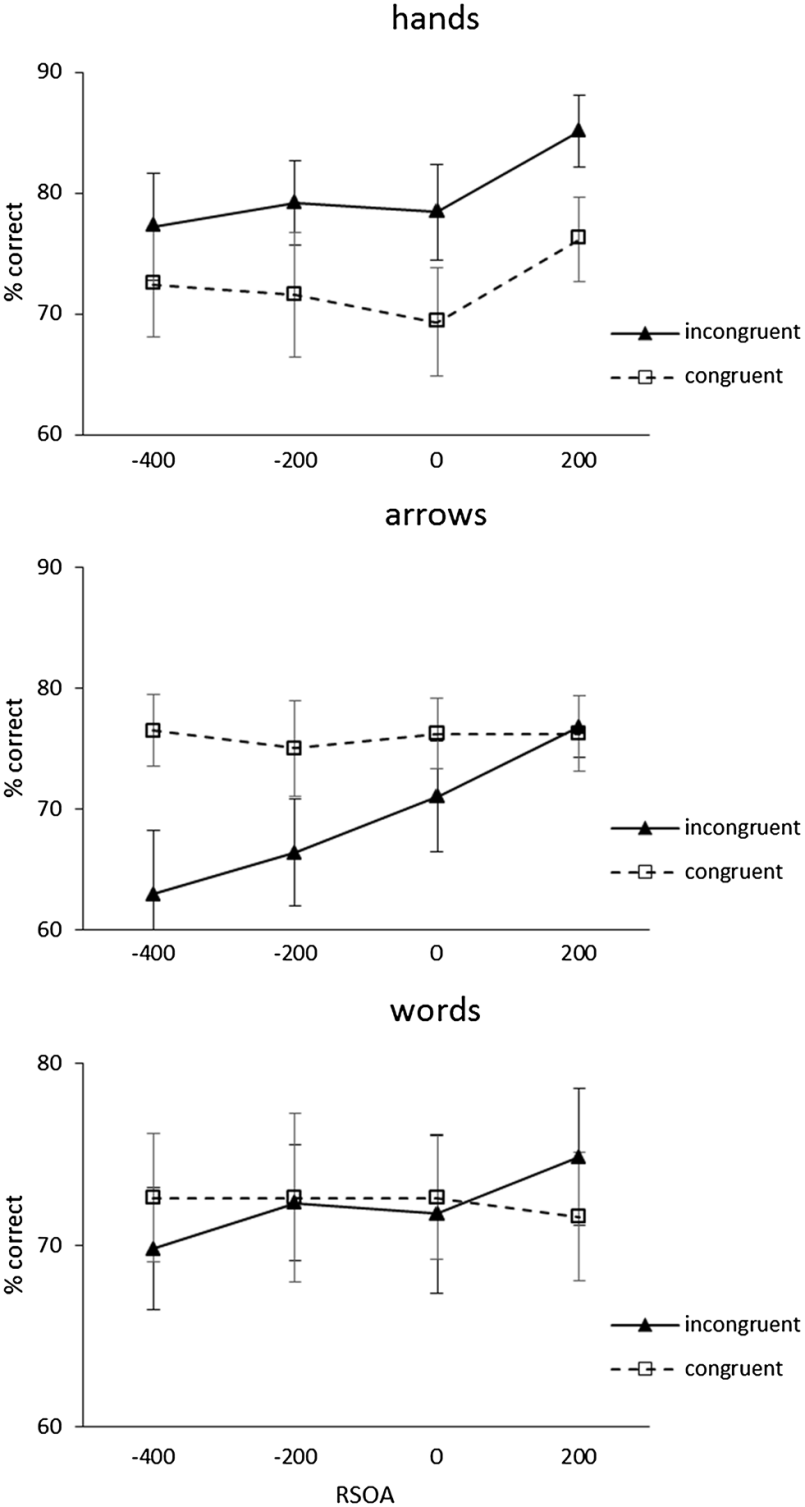
Mean accuracy for RSOA in Experiment 1. Mean accuracy is displayed in dependence on stimulus set, RSOA, and congruency. *Error bars* represent inferential confidence intervals, according to Tryon (2001)

Despite the non-significant three-way interaction, we analyzed the modulation of the priming effect by RSOA separately for the different stimulus sets, because this modulation seems to point in different directions (see Fig. 5). In two-way repeated measures ANOVAs with the factors RSOA and congruency, the factors significantly interacted only for arrows, *F*(3, 60) = 4.716, *p* = .005, *η*_*p*_^2^ = 0.191, but not for hands, *F*(3, 60) = 0.583, *p* = .628, *η*_*p*_^2^ = 0.028, or words, *F*(3, 60) = 0.774, *p* = .513, *η*_*p*_^2^ = 0.037. The interaction with arrows was due to a decrease of the positive priming effect with RSOA, while the priming effect for hands rather increased numerically with RSOA, which was however, not significant.

### Discussion

We hypothesized that all stimulus sets would show negative priming effects, and that the priming effect would get stronger with higher set-level congruency between stimulus and response. That is, the priming effect should have been stronger for hands than for arrows than for words.

Our predictions have been confirmed by the results for hand and word stimuli. First, for both stimulus set, element-incongruent trials lead to better performance than element-congruent trials, though the effect was not significant for words. Second, the magnitude of the priming effect was stronger for hands than for words. However, for arrow stimuli, the results were surprising and not predicted by our hypotheses. Indeed, a significant motorvisual priming effect was observed, but contrary to our expectancy, it was positive. This result stands in stark contrast to previous motorvisual priming studies with arrowheads (see Thomaschke, Hopkins, & Miall, 2012a, for a review).

Yet, there is a testable explanation for this unexpected result, based on the planning and control model (PCM) of motorvisual priming (Thomaschke, 2012; Thomaschke et al., 2012a). According to the PCM, there is a fundamental difference between the processing of scalar and categorical representations in motor cognition. Categorical representations code action features like the identity of a graspable object, the identity of the acting effector, the valence of the action, etc. These representations classify actions into rather coarse-grained classifications. They convey, among others, also symbolic and semantic information about actions. Scalar representations, on the contrary, code the action’s current position as coordinates in a feature space with metric properties, on dimensions like location, orientation, size, and weight. Scalar representations allow, for instance, computing the future path of actions, or its exact spatial relation to objects.

Categorical representations of action features are known to be involved in action planning and selection, whereas scalar representations are primarily involved in action control (Glover, 2004; Glover, Wall, & Smith, 2012). The PCM claims that action planning is primarily responsible for negative motorvisual priming. Selection of an action binds all representations of categorical action features into a compound representation of that action, and shields them against other cognitive processes. Thus, perception of such features is impaired during action (Hommel et al., 2001; Müsseler, Steininger, & Wühr, 2001). As action selection (not action control) is the primary explanatory domain of ideomotor theory, our literature review was focused on studies, where stimuli and responses overlapped on categorical dimensions. Accordingly, we have chosen the stimulus sets in for the present study so that they overlapped with the response on a categorical stimulus dimension (i.e., the binary categories ‘left’/‘right’). In line with all previous motorvisual priming studies (James & Gauthier, 2009; Kunde & Kiesel, 2006; Kunde & Wühr, 2004; Müsseler, Wühr, & Prinz, 2000; see Thomaschke et al., 2012a, for a review), we hypothesized that the priming effect would be negative.

However, PCM also claims that the processing of scalar representation in action control leads to positive motorvisual priming effects. Scalar representations play an important role in fast online action feedback processing during control; consequently, congruent scalar representations are facilitated. Accordingly positive motorvisual priming has been observed for response–stimulus overlap on various scalar dimensions, like size (Fagioli, Ferlazzo, & Hommel, 2007; Fagioli, Hommel, & Schubotz, 2007; Symes, Tucker, Ellis, Vainio, & Ottoboni, 2008; Wykowska, Hommel, & Schubö, 2011, 2012; Wykowska, Schubö, & Hommel, 2009), location (Collins, Schicke, & Röder, 2008; Deubel, Schneider, & Paprotta, 1998; Fischer & Hoellen, 2004; Hommel & Schneider, 2002; Koch, Metin, & Schuch, 2003; Linnell, Humphreys, McIntyre, Laitinen, & Wing, 2005; Müsseler, Koch, & Wühr, 2005), weight (Hamilton, Wolpert, & Frith, 2004), or orientation (Lindemann & Bekkering, 2009).

How does the PCM relate to the present results? Although it is well established in previous literature that arrows are typically processed categorically as symbols denoting the categories ‘left’ and ‘right’ (e.g., Müsseler & Hommel, 1997a), the arrows might have been processed via scalar representations in our study. Instead of representing and processing the arrows as conveying categorical symbolic information, participants might have encoded and processed locational information of the arrows. They might have attended only to the location of the arrows apex, instead of processing its symbolic meaning. Evaluating whether the arrow’s apex appeared on the left or right side of the decision relevant area, would have also allowed to classify its direction correctly. Thus, the left/right information of the arrows was represented scalar in the form of location information. As response–stimulus overlap on scalar dimensions leads to a positive priming effect, this assumption would be in line with the observed results.

We assume that the scalar processing of arrows was caused by the way we constructed the stimuli. Previous studies with arrows usually described the stimuli by the symbols ‘>’ and ‘<’ appearing in the methods sections. Further information about the thickness of the lines, the angle between these lines and so on is not given. Instead of using the standard font symbols, we constructed the stimuli from scratch as geometric triangles, with relatively broad arrowheads. This might have biased participants to scalar locational encoding of the left/right information.

This interpretation is strongly supported by the temporal dynamics of the priming effect. The influence of action planning typically declines over the course of an action, while the influence of action control increases. If the priming effect for hands and words was due to categorical processing in planning, while the priming effect for arrows was due to scalar processing in control, one would expect over the course of the action a decrease in the former two priming effects, but an increase in the latter one. These were exactly the dynamics observed in the present study as we changed the stimulus onset asynchronies.

Furthermore, the scalar processing of our arrowhead stimuli can be independently tested in Experiment 2, because also the Simon effect has been shown to differ in dynamics for scalar and categorical stimulus–response overlap (see below).

To conclude, for hand and for word stimuli, we confirmed our hypothesis: higher set-level congruency leads to a larger motorvisual priming effect. Yet, the arrow stimuli seem to have been processed as conveying scalar locational information. Processing of scalar information is, however, not within the scope of the ideomotor theory. Thus, our initial hypotheses do not apply to the arrow stimuli. We have modified the hypothesis for Experiment 2 accordingly (see below).

## Experiment 2

We measured the classic Simon effect with the same three stimulus sets as in Experiment 1. We hypothesized that the magnitude of the Simon effect would increase with the degree of set-level congruency. This means, that the Simon effect should be stronger for hands than for arrows than for words.

Based on the results of Experiment 1, we generated an additional hypothesis. The results of Experiment 1 suggest that the arrowheads we employed as stimuli were cognitively processed using scalar representations. Differently from previous studies with arrowheads the stimuli were processed as conveying scalar locational ‘left’/‘right’ information instead of categorical symbolic ‘left’/‘right’ information. There is corroborative evidence from a number of previous Simon-effect studies that the Simon effect with left/right stimulus locations substantially differs in many respects from such effects with other left/right representational stimuli (e.g., arrows, words, finger pictures).

The most prominent difference regards the temporal distribution of the effect. The Simon effect with horizontal location is typically large for short response times, but continually declines with slower response times (e.g., Burle, Possamaï, Vidal, Bonnet, & Hasbroucq, 2002; De Jong, Liang, & Lauber, 1994; see Dittrich, Kellen, & Stahl, 2014, for a review). In contrast, the Simon effect for left/right words (Pellicano et al., 2009), arrows (Miles & Proctor, 2012), finger pictures (Catmur & Heyes, 2011), gaze direction (Ansorge, 2003; Zorzi, Mapelli, Rusconi, & Umiltà, 2003), or objects (Cho & Proctor, 2010, 2011; Riggio et al., 2008; Fischer & Dahl, 2007), increases over time (see Proctor, Miles, & Baroni, 2011, for a review). The difference between locations and other left/right representations is further corroborated by correlational patterns (Miles & Proctor, 2012) and event-related potential measures (Cespón, Galdo-Álvarez, & Díaz, 2013).

Hence in order to confirm that the arrowheads employed in our study (Experiment 1) are processed as conveying scalar locational information, we will also analyze the time course of the Simon effect. We hypothesize that the Simon effect for hands and for words will increase over time, while the effect for arrows will decrease over time.

### Method

#### Participants

Twenty-four students from Lancaster University (18 female, 6 male) participated in this study. They received £3 or course credit. Participants had a mean age of 19.04 (SD 1.94; range 18–25). All participants were naïve with respect to the purpose of the study and classified themselves as having normal or corrected-to-normal visual acuity. None of them participated in Experiment 1.

#### Stimuli

The stimulus sets were the same hand picture, arrow, and word sets also employed in Experiment 1, with the exception that stimuli were now displayed in either blue or yellow on a black background.

#### Procedure

Participants were instructed to respond by left and right key presses to the color of the stimulus. The implicit left/right dimension of the stimuli was not mentioned in the instructions. The mapping from colors to keys was counterbalanced across participants, and remained constant throughout the experiment. The procedure consisted of three blocks, each with a different set of stimuli. The order of stimulus sets was counterbalanced across participants.

Each trial began with the target stimulus, which was visible until a response was made. In the case of a correct response, an inter-trial interval of 1 s followed. In the case of a response error, an error message was displayed in red for 3 s before the inter-trial interval. In blocks with arrow and with word stimuli, a fixation cross was displayed during the inter-trial interval. In the block with the hand stimuli, a neutral hand drawing—with none of the index fingers extended—was displayed during the inter-trial interval instead of a fixation cross.

Each block consisted of 100 trials with correct responses. There was an equal number of left and right responses, as well as an equal number of compatible and incompatible responses. The order of trials within a block was randomized. When participants responded incorrectly, the invalid trial was repeated at the end of the block, until in total 100 correct trials were completed for that block. Each block was preceded by a practice phase of three trials, and was followed by a self-paced pause. The entire procedure lasted for about 10 min.

### Results

**Fig. 6:**
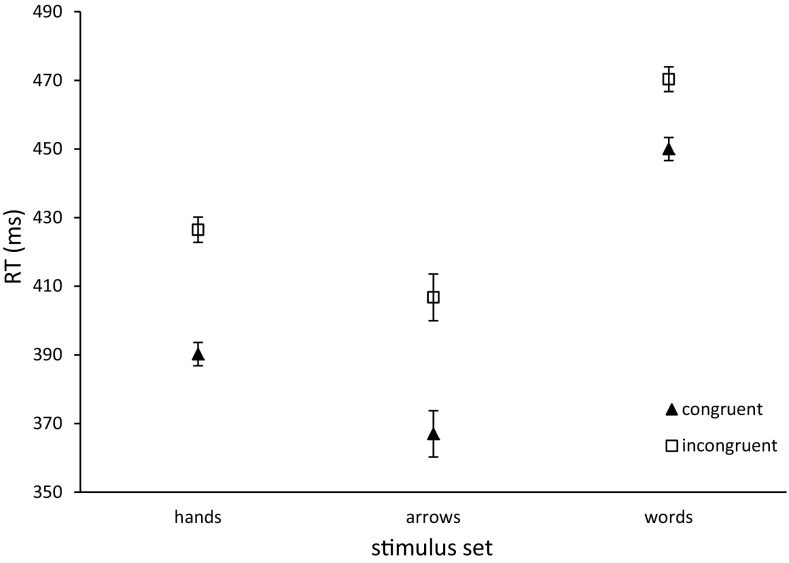
Response times in Experiment 2. Mean response times are displayed dependent on stimulus set and congruency. *Error bars* represent inferential confidence intervals according to Tryon (2001)

Mean response times in correct trials are displayed in Fig. 6, grouped according to stimulus type and congruency. In a 3 (stimulus set) × 2 (congruency) repeated measures ANOVA, the main effect of stimulus set attained significance, *F*(2, 42) = 210,81, *p* < .001, *η*_*p*_^2^ = 0.909, with faster responses to arrows, 387 ms, than to hands, 408 ms, and words, 460 ms. The main effect of congruency was also significant, *F*(1, 21) = 144, 36, *p* < .001, *η*_*p*_^2^ = 0.873, with faster responses to congruent, 402 ms, than to incongruent, 434 ms, responses. Most importantly, stimulus set and congruency did significantly interact, *F*(2, 42) = 4.88, *p* = .012, *η*_*p*_^2^ = 0.189. Post hoc comparisons revealed that the Simon effect for hand pictures, 36 ms, was significantly larger than for words, 20 ms, *t*(21) = 3.95, *p* = .001. Also the Simon effect for arrows, 40 ms, was significantly larger than for words, *t*(21) = 2.50, *p* = .021. Yet, the difference between hands and arrows was not significant, *t*(21) = 0.48, *p* = .639.

**Fig. 7:**
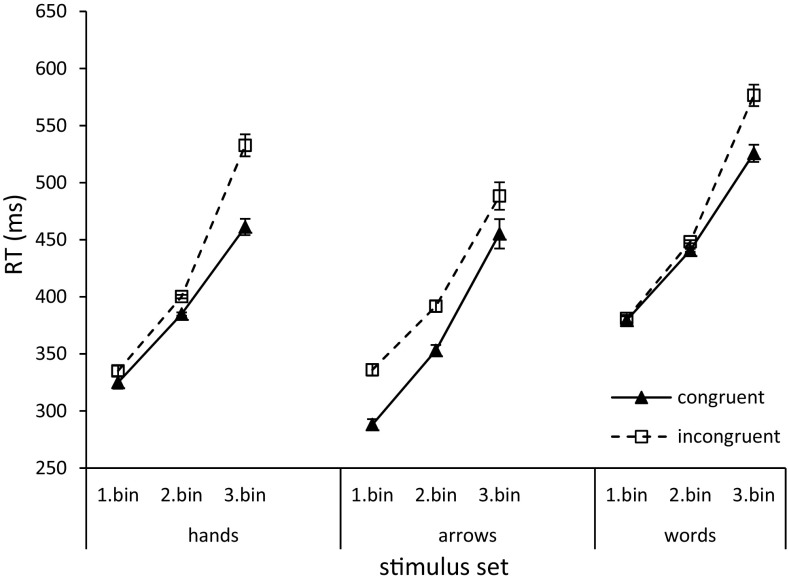
Response times in Experiment 2 for different bins. Mean response times are displayed dependent on stimulus set, bin of the response time distribution, and congruency. *Error bars* represent inferential confidence intervals according to Tryon (2001)

In order to check whether the stimulus sets have been processed by categorical or scalar representations of the left/right dimension, an additional distributional analysis was conducted. Response times for congruent and for incongruent trials were grouped into three bins, including the fastest, the middle, and the slowest third, respectively, of the response time distribution (see Fig. 7). A 3 (bins) × 3 (stimulus sets) × 2 (congruency) repeated measures ANOVA was conducted. The interaction between bin and stimulus set was significant, *F*(2, 42) = 25,45, *p* < .001, *η*_*p*_^2^ = 0.548, due to a linear trend towards higher Simon-effect magnitudes for slower bins, *F*(1, 21) = 23.75, *p* < .001, *η*_*p*_^2^ = 0.531. The three-way interaction between bin, congruency and stimulus set also attained significance, *F*(4, 84) = 20.76, *p* < .001, *η*_*p*_^2^ = 0.497.

The latter interaction was resolved by three separate 3 (bin) × 2 (congruency) ANOVAs for each stimulus set. Congruency was significant for all three ANOVAs. For hand stimuli, the interaction between bin and congruency was significant, *F*(2, 42) = 38.00, *p* < .001, *η*_*p*_^2^ = 0.644, due to a linear trend towards larger Simon effects with slower bins, *F*(1, 21) = 32.27, *p* < .001, *η*_*p*_^2^ = 0.640. For words, the interaction between bins and congruency was also significant, *F*(2, 42) = 31.828, *p* < .001, *η*_*p*_^2^ = 0.602. This interaction was in the same direction as for hands: larger Simon effects for slower bins, *F*(1, 21) = 34.84, *p* < .001, *η*_*p*_^2^ = 0.624. For arrows, sphericity was violated for the bin x congruency interaction (Mauchly’s *W* = 0.083, *p* < .001). Consequently, an analogous multivariate test was conducted (O’Brien & Kaiser, 1985). The interaction was significant *F*(2, 20) = 15,46, *p* < .001, *η*_*p*_^2^ = 0.607, but in the opposite direction as for hands: the Simon effect decreased with slower bins (see Fig. 7).

Error rates were extremely rare, *m* = 1.71%, SD = 2.39. Thus, we performed only the main ANOVA with the factors stimulus set and congruency on the mean correctness. The individual cell means and standard deviations are provided by Table 3. The main effect for stimulus set was significant, *F*(2, 42) = 7.333, *p* = .002, *η*_*p*_^2^ = 0.259, with correctness for arrowheads being superior to hands and words. The latter stimulus sets produced roughly the same percentage of errors (see Table 3). Participants were on average more correct in congruent trials than in incongruent trials, *F*(1, 21) = 20.456, *p* < .001, *η*_*p*_^2^ = 0.493, but the factors did not interact, *F*(2, 42) = 0.576, *p* = .566, *η*_*p*_^2^ = 0.027.

**Table 3:** Mean accuracy rates for each combination congruency, and stimulus set in Experiment 2

Stimulus set	Congruent	Incongruent
Hands	98.89 (1.63)	96.94 (3.04)
Arrows	99.82 (0.58)	98.67 (1.52)
Words	98.76 (1.51)	96.96 (3.50)

SDs are displayed in parentheses. Values are rounded to two decimal places

### Discussion

First, we predicted that stimulus sets with higher set-level congruency to the response set would produce larger Simon effects. This prediction was confirmed by the results for hand pictures and for words. Hand pictures, having high set-level congruency with key presses, produced a larger Simon effect (36 ms) than did word stimuli (20 ms) with lower set-level congruency. However, the results for arrows were not so clear: we expected arrows to be somewhere in the middle between hand pictures and words with regard to Simon-effect magnitude, because their set-level congruency is lower than for pictures, but higher than for words. This was only partly confirmed: the Simon effect for arrows (40 ms) was larger than for words, but not significantly different from hand pictures.

Second, we predicted that the Simon effect for hands and words would increase, while the effect for arrows would decrease. This prediction was fully confirmed. This pattern supports our conclusion from the results of Experiment 1, namely that our arrowhead stimuli were processed by scalar location representation, instead of symbolic or categorical representations.

## General discussion

### Summary of results

We predicted that the strength of both visuomotor and motorvisual priming would be modulated in the same direction by the set-level congruence between stimulus and response set. This prediction has been confirmed with two out of three employed stimulus sets. Hand pictures have higher set-level congruence with key presses than word stimuli. Consequently, visuomotor as well as motorvisual priming were both more pronounced with hand stimuli than with word stimuli. This supports ideomotor theory’s previously untested prediction that the visuomotor and motorvisual priming are mediated by the same representational mechanism.

For the third stimulus set, consisting of left and right arrows, several aspects of the results patterns in both experiments show that the response-congruent/incongruent visual information was represented in a scalar location-based format. However, our hypothesis about set-level congruence only applies to categorical representation, because scalar representations are not processed by action selection—the main explanatory domain of ideomotor theory. Thus results from this stimulus set contribute no evidence for or against our hypothesis.

### Arrows in planning and control

The incidental findings with the arrow stimuli can be seen as support for the PCM (Thomaschke et al., 2012a). The PCM postulates that categorical representational processing in action planning is responsible for negative motorvisual priming effects, and that scalar representational processing in action control is responsible for positive motorvisual priming effects.

These assumptions are supported by the temporal effect dynamics in both experiments. For hand and word stimuli, the dynamics of the Simon effect indicated a categorical representational format, and the dynamics of the motorvisual priming effect indicated processing for action planning. Consequently, both stimuli showed a negative motorvisual priming effect. For the arrow stimuli, the dynamics of the Simon effect indicated scalar representational format, and the dynamics of the motorvisual priming effect indicated processing for action control. Consequently, a positive motorvisual priming effect was observed for the arrow stimuli.

An alternative explanation for the positive motorvisual priming effect with arrow stimuli would propose that the effect was actually indeed a negative priming effect based on categorical left/right representations, but that the interpretation of the symbols as left and right was reversed. Stevanovski, Oriet, and Jolicoeur (2002) have shown that instructions can reverse the negative priming effect for arrows, when, for example, the symbol < is described as a headlight pointing to the right, instead of an arrow pointing to the left. However, we prefer the interpretation as a positive priming effect, for three reasons. First, we instructed the participants to say whether the arrow pointed left or right, making the symbols’ directional interpretation unambiguous. Second, the temporal dynamics of effects in both experiments independently suggest that the left/right information conveyed by the arrows was processed as scalar location information. Third, the same arrow symbols figured as cues for the motor response in all three conditions. This has been common practice in previous motorvisual priming studies (Müsseler & Hommel, 1997a, b; Thomaschke et al., 2012b; Wühr & Müsseler, 2001; see Stevanovski, Oriet, & Joliecœur, 2003, for a discussion). Assigning the stimuli, in their role as cues, to left and right responses, strongly suggests that they are interpreted in the same direction in their role as discrimination targets.

However, our findings suggest that the frequently employed left/right symbols < and > are not always automatically processed in a symbolic categorical way. Subtle aspects of symbol construction may lead to processing the conveyed left/right information in scalar form, by processing the locations of the arrow’s peaks. This might pose an alternative explanation for some previously unaccounted findings: the great majority of motorvisual priming studies with arrows found negative priming effects (see Thomaschke, 2012, for a review), but occasionally reversed effects have been observed (e.g., Caessens & Vandierendonck, 2002; Fischer, 1997). Scalar processing of arrowheads, due to idiosyncrasies in stimulus construction, could potentially account for these exceptions.

### Other priming designs

Our study used only two types of experimental design to assess visuomotor and motorvisual priming effects. We briefly review how these might be related to other designs. On the visuomotor side, we focused on designs where the priming information was irrelevant to the task, the so-called Simon effect. However, ideomotor theory does also apply to designs where the priming information is task-relevant, typically referred to as stimulus–response compatibility effects. In these designs, the stimulus response mapping is either compatible (e.g., responding with left key to a left pointing arrow, and with a right key to a right pointing arrow) or incompatible (e.g., responding with left key to a right pointing arrow, and with a right key to a left pointing arrow; see Proctor & Vu, 2006, for a review). Responses in blocks with compatible mapping are typically faster than with incompatible mapping (Donders, 1868; Fitts & Deininger, 1954).

This effect is also commonly explained in terms of ideomotor theory, as stemming from the overlap of representational codes between perception and action (Kornblum, Hasbroucq, & Osman, 1990; Kornblum & Lee, 1995). Several previous studies have investigated the relation between set-level and element-level compatibility in the stimulus–response compatibility paradigms. The results are in general in line with the finding in Simon-effect studies reviewed in the introduction: compatibility effects are larger for stimulus and response sets with high set-level compatibility than with low set-level compatibility (Lameira, Pereira, Fraga-Filho, & Gawryszewski, 2015; Proctor, Wang, & Vu, 2002; Wang & Proctor, 1996). However, there is no previous study comparing the effect of set-level compatibility on the stimulus–response compatibility effect and on the motorvisual priming effect with each other. Based on the present results, we hypothesize that there would be an analogous manipulation of set-level compatibility for stimulus–response compatibility and for motorvisual priming.

With regard to motorvisual priming we choose a dual-task design, because it is by far the most common paradigm in the literature on motorvisual influences. However, in some studies motorvisual priming is investigated by single-task experiments with prepared responses. In the prepared response paradigm, the go-signal for an already prepared response is either congruent or incongruent to the response. Congruency effects are typically explained by priming from the prepared response on visual processing of the go-signal (Craighero, Bello, Fadiga, & Rizzolatti, 2002; Craighero et al., 1998; Craighero, Fadiga, Rizzolatti, & Umiltà, 1999; Grosjean & Mordkoff, 2001; Lindemann & Bekkering, 2009). Yet, motorvisual priming in the prepared response paradigm has to our knowledge only been investigated with scalar stimulus response overlap (i.e., location or orientation).

Concerning stimulus modality, our study was focused on visual perception. However, visuomotor and motorvisual priming phenomena have also been described for other modalities, like audition (Repp & Knoblich, 2007, 2009; Yon, Edey, Ivry, & Press, 2017), or tactile perception (Juravle, Binsted, & Spence, 2017; Juravle & Deubel, 2009; Juravle, Deubel, Tan, & Spence, 2010; Juravle, McGlone, & Spence, 2013). As those priming phenomena can also be explained by ideomotor processing, we assume our conclusions to generalize to other modalities.

### Conclusions

Visuomotor and motorvisual priming are both modulated by set-level congruency in the same way: stronger effects with higher set-level congruency. This pattern was confirmed in two experiments with hand and word stimulus sets. These results support the ideomotor theory, which assumes that visuomotor and motorvisual priming are both due to shared representations between perception and action.

Above that we unexpectedly observed a positive motorvisual priming effect with arrowhead stimuli. We explain this effect by scalar representations in the processing of the arrowhead stimuli. Effect dynamics in both experiments corroboratively support this explanation. Thus, we conclude that arrowheads are not always processed in a categorical fashion.
